# Politics by Automatic Means? A Critique of Artificial Intelligence Ethics at Work

**DOI:** 10.3389/frai.2022.869114

**Published:** 2022-07-15

**Authors:** Matthew Cole, Callum Cant, Funda Ustek Spilda, Mark Graham

**Affiliations:** Social Sciences Division, Oxford Internet Institute, University of Oxford, Oxford, United Kingdom

**Keywords:** artificial intelligence, labor, work, ethics, technological change, collective bargaining, industrial relations, job quality

## Abstract

Calls for “ethical Artificial Intelligence” are legion, with a recent proliferation of government and industry guidelines attempting to establish ethical rules and boundaries for this new technology. With few exceptions, they interpret Artificial Intelligence (AI) ethics narrowly in a liberal political framework of privacy concerns, transparency, governance and non-discrimination. One of the main hurdles to establishing “ethical AI” remains how to operationalize high-level principles such that they translate to technology design, development and use in the labor process. This is because organizations can end up interpreting ethics in an *ad-hoc* way with no oversight, treating ethics as simply another technological problem with technological solutions, and regulations have been largely detached from the issues AI presents for workers. There is a distinct lack of supra-national standards for fair, decent, or just AI in contexts where people depend on and work in tandem with it. Topics such as discrimination and bias in job allocation, surveillance and control in the labor process, and quantification of work have received significant attention, yet questions around AI and job quality and working conditions have not. This has left workers exposed to potential risks and harms of AI. In this paper, we provide a critique of relevant academic literature and policies related to AI ethics. We then identify a set of principles that could facilitate fairer working conditions with AI. As part of a broader research initiative with the Global Partnership on Artificial Intelligence, we propose a set of accountability mechanisms to ensure AI systems foster fairer working conditions. Such processes are aimed at reshaping the social impact of technology from the point of inception to set a research agenda for the future. As such, the key contribution of the paper is how to bridge from abstract ethical principles to operationalizable processes in the vast field of AI and new technology at work.

## Introduction

The advent of a new era of innovation in machine learning AI and its diffusion has prompted much speculation about how it is reshaping society (Gentili et al., 2020). As well as seeing it as an opportunity to advance common social goals, many have also identified how such developments may pose significant risks, particularly to actors who are already disempowered and discriminated against. Consequently, much thought has gone into the risks and opportunities of AI, and the creation of principles for its ethical development and deployment. However, this thought tends to be at the intersection of the instrumental-economic and abstract ethics (Algorithm Watch, 2020), with operationalization generally restricted to the domain of privacy concerns, transparency and discrimination. Questions around workers' fundamental rights, job quality (see Cazes et al., 2015) and working conditions more generally have not received as much attention.

Given that technologies tend to be path-dependent (MacKenzie and Wajcman, 1999), embedding a set of concrete principles and benchmarks from the outset of technological diffusion is an important way to control their social effects as it supports regulation. There is an urgent need to create a set of evaluation mechanisms that directly address the impact of AI on working conditions, and that can feed into regulation of these technologies. However, research on this topic is limited. A Scopus query for the term “AI ethics” retrieves 2,922 results. When “work” is added to the search string, this number drops by more than half, to 1,321 results. Of these, 309 are publications in the social sciences, indicating limited engagement of our field with the topic. When analyzed in detail, we see that only 58 of them discuss AI ethics pertaining to work and employment. Most of these focus on digital wellbeing (Burr et al., 2020) or worker wellbeing (Nazareno and Schiff, 2021), the impact of algorithms on decision-making in government, employment agencies (Kuziemski and Misuraca, 2020), predictive policing (Asaro, 2019; Yen and Hung, 2021) and bias in algorithmic decision-making (Hong et al., 2020; Metaxa et al., 2021). The studies that are specifically on work and employment target recruitment (Yam and Skorburg, 2021), human resources management (Bankins, 2021) or workplace management (Jarrahi et al., 2021). There is, thus, a clear gap in the literature concerning how AI ethics relates to fairness, justice and equity in the context of work and employment.

Against this background, this paper sets out a critique and a research agenda to address this gap. However, the pathway from high-level principles to enforceable regulation on working conditions has yet to be clearly defined. As noted by Wagner (2018, 2019), the current focus on AI ethics is simply a watered-down version of regulation—especially when technology companies opt for voluntary codes of practice that they've shaped themselves. As Algorithm Watch (Thiel, 2019) notes, most existing AI ethics guidelines are non-binding, and they operate on an opt-in basis. AI ethics can therefore be something companies congratulate themselves on for their good intentions, without the need to turn these so-called ethics into actual practice. Hence, there is an urgent need to move from abstract principles to concrete processes that ensure compliance. This is a necessary step, irrespective of emerging regulations on the issue.

In this article, we provide a critique of how AI systems are shaping working conditions before identifying ways in which it can foster fairer work (see Section Proliferating Principles). We first review a selection of AI guidelines, ethics and meta-analyses using Boolean search, and outline four critiques that cut across the recent proliferation of ethical guidelines. These are summarized by the four headings: (1) Not everything is a trolley problem (ethical critique); (2) AI is not that special (socio-technical critique); (3) The problem with automatic politics (ethico-political critique); (4) Big Tech Ethics is Unilateral (a socio-political critique). These critiques set up methodological basis for the University of Oxford's AI for Fairwork project (supported by the Global Partnership on AI), which aims to produce a set of AI guidelines that avoid these pitfalls and contribute to fairer uses of AI at work. These guidelines, a draft of which are presented in Section Proposed AI for Fairwork Standards below, are not exclusively intended to assist in either risk mitigation or opportunity maximization. Instead, we view those two goals as inseparably linked. By shifting our attention from mere negative outcomes of technological development to the processes of technological innovation and design, we aim to embed fairness into the very technologies that get built, instead of attempting to fix problems once and as they arise.

We position our understanding of fairness as both as an ethical absolute that should be strived for, but also as a virtue that is context dependent to time, space and conditions. As such, we treat fairness not as a static and unchanging category or end point in itself, but rather as a process that involves continual revision relative to material circumstances. To use the language of Silicon Valley: making things fairer is an iterative process. Agents are required to constantly attempt to move toward a horizon of fairness that they can't quite reach. This will likely be the case for a long time to come, as we can foresee no final point at which any work environment could be declared completely fair—at least, not under this economic and political system.

## From Ethics to Fairness

### Proliferating Principles

The proliferation of real (or speculative) AI use-cases and corresponding national industrial strategies (HM Government, 2021), has provoked a swath of voluntarist ethical guidelines from an array of actors, from the OECD to the European Parliament, Microsoft, and even the Pope. Governments, supra-national institutions and non-governmental organizations have all shown an interest in understanding and regulating AI systems. In this section, we review a selection of such principles that are most relevant to our research and the development of Fairwork principles for AI. By investigating a selection of these principles more closely, we can lay the groundwork for our subsequent critique.

The OECD (2019) Principles on Artificial Intelligence were the first AI ethics guidelines signed up to by governments. They complement existing OECD standards in areas such as privacy, digital security risk management, and responsible business conduct (see Table 1). The G20 also adopted “human-centered AI principles” that drew on these principles. In a similar vein, the European Parliament (European Parliament, 2019) has drawn up a code of voluntary ethics guidelines for AI and robotics engineers involving seven key requirements (see Table 1). Such requirements informed the 33 policy and investment recommendations that guide the proposal for “Trustworthy AI” put forward by the EU High-Level Expert Group on Artificial Intelligence (AI HLEG) and their self-assessment checklist (High-Level Expert Group on AI, 2020). The European Commission wants “Trustworthy AI” that puts “people first” (European Commission, 2020). However, the EU's overall approach emphasizes the commercial and geopolitical imperative to lead the “AI revolution”, rather than considering in detail the technological impact on workers and work. It has been noted that members of the AI HLEG have already condemned the results as an exercise in ethics washing (Metzinger, 2019).

**Table 1:** Summary of four illustrative AI principles.

**Principle author**	**Stated values**	**Specific discussion of work**
OECD	(1) Regular engagement of multiple external and internal stakeholders; (2) mechanisms for independent oversight; (3) transparency around decision-making procedures; (4) justifiable standards based on evidence; (5) clear, enforceable legal frameworks and regulations.	Affirms the importance of international labor rights. Suggests that workers should be aware of their interactions with AI systems. Encourages governments to prepare for “labor market transition” through skill development social dialogue, and promoting increases in safety and job quality.
UNESCO	(1) Proportionality and “do no harm”; (2) safety and security, fairness and non-discrimination; (3) sustainability, right to privacy and data protection; (4) human oversight and determination; (5) transparency and explainability; (6) responsibility and accountability, awareness and literacy; (7) multistakeholder and adaptive governance and collaboration.	Encourages governments to implement impact assessments that monitor, amongst other things, the effect of AI on labor rights, Strongly emphasizes the need for skill development, retraining and “fair transition” for at-risk employees. States the need for ongoing research on the impact of AI systems on work.
European Parliament	(1) Human agency and oversight; (2) robustness and safety; (3) privacy and data governance; (4) transparency; (5) diversity, non-discrimination and fairness; (6) societal and environmental well-being; (7) accountability	Notes concern about impact on labor market and describes workers as one of nine relevant stakeholder groups.
President of the United States	(1) Lawful and respectful of our Nation's values; (2) purposeful and performance-driven; (3) accurate, reliable, and effective; (4) safe, secure, and resilient; (5) understandable; (6) responsible and traceable; (7) regularly monitored; transparent; (8) accountable.	None.

Following this trend, UNESCO's 2021 Recommendation on the Ethics of Artificial Intelligence' also emphasize the production of “human-centered AI” around 10 principles. UNESCO also proposes a set of 11 policy areas aligned with these fundamental principles for member states to consider. The President of the United States issued an “Executive Order on Promoting the Use of Trustworthy Artificial Intelligence in the Federal Government”, that provided “Principles for the use of AI in Government” (White House, 2020).

The US executive order was likely in response to the Algorithmic Accountability Act proposed on April 10, 2019 in the United States Congress, which aimed to legislate rules for evaluating highly sensitive automated systems. The Act was never taken to a vote, but a new version has recently been introduced on March 1, 2022 that aims “To direct the Federal Trade Commission to require impact assessments of automated decision systems and augmented critical decision processes, and for other purposes” (United States Congress, 2022). This legislation aims to increase certain kinds of transparency with regard to automated decisions affecting US citizens from the use to algorithms. It requires both the firm that builds the technology enabling the automation as well as the company using it to make the decision to conduct impact assessments for a range of factors including bias, effectiveness, and security. Furthermore, the bill aims to provide a benchmark requirement which stipulates that companies assess the impacts not only of new automated decision-making processes, but also already-existing ones. It mandates that the Federal Trade Commission (FTC) creates regulations that standardizes assessment and reporting, requires auditing of impact-assessment and the FTC to publish an annual anonymized report on trends and provide a public dataset of automation decision technologies.

Ethical principles have proliferated to such a degree that there are now multiple databases cataloging them online. One such inventory of AI Ethics Guidelines is crowd sourced and maintained by the NGO Algorithm Watch. The database currently identifies 173 sets of principles “of how systems for automated decision-making (ADM) can be developed and implemented ethically”. These fall into three broad categories: binding agreements (8), voluntary commitments (44), and recommendations (115). Similarly, the OECD maintains a live database showing over 700 initiatives related to AI policy from 60 countries, territories and the EU.^1^ In a recent study, Jobin et al. (2019) identified 84 different ethical AI standards, produced by a range of private companies, government agencies, research institutions, and other organizations. They identified 11 overarching principles, namely (in order of popularity): transparency, justice and fairness, non-maleficence, responsibility, privacy, beneficence, freedom and autonomy, trust, dignity, sustainability, and solidarity. Only transparency, justice and fairness, non-maleficence, responsibility, and privacy appeared in most of the standards.

These various efforts to track the ongoing proliferation of guidelines is a useful starting point for thinking about how effective they might be in practice. Mittelstadt et al. (2016) identified six key issues raised by the use of algorithms (which they define in such a way as to include much of what we might call AI): inconclusive evidence, inscrutable evidence, misguided evidence, unfair outcomes, transformative effects, and traceability. These concerns appear to have stayed relatively consistent over time (Roberts et al., 2021), which is somewhat problematic given the limited set of stakeholder perspectives contained in the field of principles (Hickok, 2021). We could summarize that the last 5 years have seen the same people raising the same issues, with limited evidence of progress or widening participation in the discussion.

To these concerns about perspectival limitations, we would also add concerns about the ideological limitations of these principles. The current debate on AI ethics in the literature tends to be limited by ontological, epistemological and political assumptions drawn from classical liberal thought—namely around rights and privacy. The horizon of these principles takes certain conditions as given: private ownership of means of production, capitalist social relations, the institutional reproduction of such relations, the individualist perspective on decision-making and responsibility and the embeddedness of technologies within this context. As a result, many participants in the debate have only been able to consider courses of action which fall within these limitations. An example of how this limited frame raises problems is Oren Etzoni's (2018) “Hippocratic oath for artificial intelligence practitioners”. The oath—an attempt to model a framework for AI ethics analogous to that underpinning the medical profession— reads:

*I will consider the impact of my work on fairness both in perpetuating historical biases, which is caused by the blind extrapolation from past data to future predictions, and in creating new conditions that increase economic or other inequality*.

But as Mittelstadt (2019) argues: the lack of an analogous institutional context to medicine means that the Hippocratic principle-based model of ethical regulation doesn't map well to AI. Indeed, AI development lacks “common aims and fiduciary duties, professional history and norms, proven methods to translate principles into practice, robust legal and professional accountability mechanisms” (Mittelstadt, 2019, p. 1). This problem with simply mapping one domain onto another—and assuming it will work in that new context—points to a broader concern with the impact of guidelines, particularly in the context of working life.

The central question here is how to translate ethical principles into ethical practice (Hagendorff, 2020, 2021). The difficulty of providing a robust answer has been repeatedly identified. Hagendorff (2020) examines whether certain principles have a real-world impact on the ethics of process and outcomes in AI-mediated work and concluded “No, most often not” (Hagendorff, 2020, p. 99). Floridi (2019) identifies risks in the transition from *what* to *how* that include: digital ethics shopping, “bluewashing” (i.e., digital greenwashing), lobbying, ethics dumping (outsourcing to other actors), and shirking. Morley et al. (2021) argue that, while principles are important in defining the normative values against which AI is evaluated, the translation of broad principles into concrete action is difficult. Following the metaphor of cloud computing, they envision a hybrid institutional arrangement that can offer “ethics as a service”.

Despite the breadth and depth of work on AI ethics, there remains a profound blind spot in terms of implementation, since organizations are left largely to interpret and enact ethical guidelines themselves and then assess if they are abiding by them. This exposes workers to potential abuse of AI technologies not only in terms of digital Taylorism, but also the degradation of work by reproducing biases and inequalities, intensifying work and denying collective control. Mitchell (2019), (p. 152) highlights the diversity of ethical issues vying for the attention of regulators:

*Should the immediate focus be on algorithms that can explain their reasoning? On data privacy? On robustness of AI systems to malicious attacks? On bias in AI systems? On the potential “existential risk” from superintelligent AI?*

Yet questions of work and employment are conspicuously absent from both this set of questions and the ethics guidelines mentioned above. Indeed workers and employees are rarely cited when lists of relevant AI stakeholders are listed.

While some legislation relating to AI transparency in the workplace has been passed in certain countries such as Spain and France, further steps are needed to ensure that laws and requirements of this type are enforceable and effective (Algorithm Watch, 2020). Marx [1887] (1976) referred to the sphere of production as “the hidden abode” in order to point out how the purported values of liberalism were restricted to operating only in the market. So far, the field of AI ethics has, by and large, also refused to venture into this black box. Rather than deal with the contentious social relations which structure production and the workplace, the current debate so far has focused its attention on how AI impacts its users in their roles as citizens and consumers, but not as workers.

### What Is “Fair” Anyway?

From Aristotle to Rawls, from Fraser to Nussbaum and Sen, fairness and its broader counterpart, justice, have acquired multiple meanings when seen from different philosophical standpoints and in different practical contexts. In all these different interpretations, however, issues of justice emerge in circumstances of scarcity, when there are then potentially conflicting claims to what each person is entitled to, or how institutions can administer fair allocation of resources (Miller, 2021). Thus, fairness for whom, and fair according to what/whose criteria remain as two key questions. In other words, we would not need fairness or justice, if we had unlimited resources and as individuals we had unlimited skills and capabilities. We need fairness and justice because there are limited resources and as humans we have limited capacities (individually). Following from this, in answering how to be a virtuous person for instance, Aristotle counts justice as one of the four seminal virtues a person should have, and notes that it is thought to be “another's good” because it is defined always in relation to another individual, another status and positionality, and as such he conceptualizes justice as proportionality (Aristotle, 2000, p. 73).

Rawls' theory of justice, which remains by far the most referenced theory on the topic, aims to solve the dilemma of establishing justice in a society where different individuals are all seeking to advance their own interests (e.g., utilitarian, modern, capitalist, and so on). While ultimately Rawls tries to reconcile the freedom of choice for individuals with fair outcome for all (as in a world of scarce resources, the choices of individuals may not always be guaranteed), Rawls presents two informational constraints for individuals in making that choice. He imagines individuals behind a “veil of ignorance” that deprives them of any knowledge of personal characteristics which might make some of the choices more available, more favorable or more easily attainable for some individuals. This ignorance of personal characteristics, skills and capabilities ultimately serves to make individuals base their choices on an impartial principle of what would be fair for everyone. Here, Rawls also suggests that this impartiality can be benchmarked by assuming one must make a choice for the worst off in society. This person, in a hypothetical context, can be the individual making the choice for others (Rawls, 1993).

For Rawls, then fairness implies some level of equal distribution in society of opportunities and resources. Scanlon (1998) argues that individuals will never realistically be able to perform a veil of ignorance because we are all aware of our own relative positions, wants and needs. Instead, he argues for a theory of justice which no one could reasonably reject, even when they are given a right to veto, should they see it as unfair. Philosophers have rightly commented that giving everyone a right to veto will ultimately create a deadlock as anyone can reject a principle which does not treat them favorably (Miller, 2021). However, Scanlon emphasizes that this will not be the case, if the principle of reasonable rejection applies, as individuals will be able to weigh if the current principle seems unfair, if an alternative would involve someone else fairing worse still (Miller, 2021). Scanlon also notes that the right to veto is significant for individuals because if a principle treats them unfairly, such as faring well for some but not others and for arbitrary reasons; individuals should be in a position to reject this (Miller, 2021), unlike in Rawls' theory, whereby individuals would not be in a position to judge whether arbitrariness played any role in individuals' decisions.

In contrast to Rawls and Scanlon, who both argue for a contractual theory of justice, Sen, for instance proposes a more distributive form of justice with the capability approach, explaining that what we need is not a theory that describes a utopian ideal of justice, but one that helps us make comparisons of injustice, and guide us toward a less unjust society (Robeyns and Byskov, 2021). In this regard, Sen (and also to an extent Nussbaum) proposes that the intention of a theory of justice is not necessarily to identify and only aim for the ideal of fairness, but rather identifying and then equipping individuals with the capabilities they would need to strive for lesser injustice, and less unfair societies. Some philosophers have argued that the capability approach overcomes some of the inflexibilities inherent to Rawlsian (or indeed Aristotelean) theories of justice, because it takes into account the different needs, circumstances and priorities of different individuals (Robeyns and Byskov, 2021).

In this paper, we define fairness not by its unchanging absoluteness, but conditionality, contextuality and proportionality based on the circumstances of individual and institutional decision-makers. In this regard, fairness influences the whole decision-making process from ideation to development and execution of AI-based systems, rather than one final goal that can be achieved once and for all. Hence, we focus more on increasing individual and organizational capabilities to guide us toward a less unfair society.

Generally, individuals will bring their own expectations to bear on the meaning of fairness, such that two people may consider the same set of working conditions fair or unfair. In order to overcome such confusion, we use “fair” in the sense of the capability approach outlined above. At the abstract level, we define fairness as direction of travel toward a more just society. When power asymmetries are being undermined through democratization, when opportunities and outcomes are being equalized, when access to self-determination and positive freedom are being opened to a wider range of people, then we consider work to be getting fairer.

Concretely, standards and benchmarks of fairness have a significant role to play as waypoints along this journey. While what qualifies as decent or good quality work can vary between and among different workers, stakeholders and policy-makers, most standards (from the ILO to OECD and Eurofound) involve six key dimensions of job quality: pay and other rewards; intrinsic characteristics of work; terms of employment; health and safety; work–life balance; and representation and voice (Warhurst et al., 2017). In this regard, we begin from the baseline standards of decent work developed by the Fairwork Project, which include fair wages, conditions, contracts, management and representation (Heeks et al., 2021). These principles have evolved over years of action-research and broadly align with the wide-range of job-quality metrics in contemporary academic research.

## Four Critiques of Artificial Intelligence Ethics

### Not Everything Is a Trolley Problem (Ethical Critique)

Current AI ethics approaches present a mix of various schools of thoughts in ethics. Sometimes we find schools that have long been in conflict with one another combined to suit the needs of the particular parties who are building the principles. The two most common schools are *consequentialist ethics* (a version of which is utilitarian ethics) and *deontological ethics*. Consequentialist ethics examines the consequences of ethical decisions and asks the ethical agent to make an ethical judgment based on the consequences that are important to her (Sen and Williams, 1982). Utilitarianism (following Bentham) suggests that the most ethical decision would be the one that provides the greatest utility for the greatest number of people. Of course, defining both “utility” and a “number of people affected” are both complex questions. In contrast, deontological ethics disregards the consequences of any ethical decision or the intentions that lead to it, but focuses entirely on the principles instead (Anscombe, 1958). Principles such as “Thou shall not kill” hold irrespective of individual circumstances and particular intentions of the ethical agent. Finally, virtue ethics (stemming from Aristotle's *Nichomachean Ethics)* argues that the only road to *eudaimonia* (or personal happiness, *flourishing*) is through living in accordance to fulfilling one's virtues (Annas, 2006).

Much of the current ethical thinking with respect to AI ignores the important differences between consequentialist, deontological and virtue ethics, and instead follows a mix and match approach, as it fits the questions and desired outcomes. Most commonly, consequentialism mixed with a touch of deontological ethics based on the assumption of a virtuous actor (e.g., developer, entrepreneur, and investor) in the field of AI is imagined and proposed. In this imaginary, the ethical proposition is done in a way that it does not conflict with or hinder the intention to “move fast and break things” (Ustek-Spilda, 2018).

Consequentialism dominates the discussions also because, in comparison to de-ontological ethics or virtue ethics, it can be seemingly easily translated to decisions that are taken in technology development. This suggests that when the consequences of a decision cannot be predicted fully, then the best option is to hope that the principles that guide that process will avoid the worst possible outcomes. We might very well ask: “worst for whom” and “worst in accordance to what criteria”? Note that this is not a call for subjectivism—that is, the ethical position that all values change from person. To person and there are no objective or absolute values, but simply to note the serious need for identifying how principles can facilitate ethical decision-making, amidst this uncertainty.

For example, the “trolley problem” is used to unpack particular issues identified in AI ethics. This thought experiment concerns a runaway trolley that will kill someone—but where a person can choose between alternative outcomes. In the version developed by MIT Media Lab, the person thinking through the problem is asked to choose between killing young children or the elderly, a small number of disabled people or a higher number of able-bodied people, an overweight person or a fit person.^2^ The problem is that by adapting this thought experiment to the AI development context, it simplifies complex decisions into either/or questions. It doesn't allow any room for the possibility of *no one* (for example) being killed. So, there is no room to discuss one of the central questions with AI—whether or not it should actually be built in the first place. Or should a problem which can be fixed with AI, actually be fixed with AI, or should it perhaps not be fixed at all (Penn, 2021).

There are many examples of AI reproducing and/or amplifying patterns of social discrimination, and the thinking used in the trolley problem being extended to solving these problems too. In 2018, for instance, Reuters published a story revealing that Amazon had been forced to scrap an AI recruiting tool that was intended to analyze CVs and score applicants from one to five. Since the tool was trained using training data taken from the hiring process at Amazon over the last 10 years, it faithfully reproduced the bias against women it found therein (Dastin, 2018). Other famous cases of discriminatory AI such as the ProPublica investigation into racial bias and the COMPAS risk assessment software used in bail, probation and sentencing decisions across the US (Angwin et al., 2016) have demonstrated the potential of serious social harms from automated discrimination. However, what is notable in many discussions of these cases is that they focus on how to design *better* hiring and risk assessment software—rather than asking if decisions of this kind should be automated at all. In the workplace context, the failure to ask serious questions about the ethical integrity of decisions to deploy AI can lead to very significant negative consequences for workers. The complexity of questions regarding issues like hiring demands more from us than a mishandled application of the Trolley Problem, or ethical theories being thrown in together just to fit in with the desired framework and outcome of a particular AI ethics project.

### AI Is Not That Special (Socio-Technical Critique)

Since its inception by John McCarthy in 1956, the academic field of Artificial Intelligence has been premised on the creation of a software program that can solve not just one narrow kind of problem, but that can apply its capacity for calculation to any kind of problem (Wooldridge, 2021). Such a truly general AI does not currently exist. While the most advanced forms of AI created to date, such as GPT-3 and AlphaGo, can outperform humans in some very limited tasks, they still have near-zero general applicability, and lack the ability to think in a manner which at all reflects the human brain (Chui et al., 2018).

Despite this, AI is often treated as an *exceptional* technology with a universalist or unbounded horizon. Indeed, despite not yet having achieved real AI, the assumption among many is that that is the direction of travel. So, rather than treating AI as a technical field concerned with advanced, non-symbolic, statistical methods to solve specific, bounded problems (facial recognition, natural language processing, etc.), AI positivists identify the field as something unprecedented. AI ethics therefore begins to become orientated toward a hypothetical future scenario, rather than the reality of our present moment.

2012 marked the start of a sea change in how AI practitioners go about their work, and it was enabled by increases in the volume of data, dataset-creating labor and computing power available for the development of AI (Cole et al., 2021). From natural language to game playing and visual object recognition, the turn to deep convolutional neural network and machine learning has allowed for significant progress across the various subfields that make up AI and is the basis for the latest surge in funding and media attention. Justified celebration of these developments has gone hand-in-hand with unjustified hyperbole about the future. In 2017, Ray Kurzweil, Google's Director of Engineering, famously claimed that the “technological singularity” would be achieved by 2045, as we “multiply our effective intelligence a billion-fold by merging with the intelligence we have created” (Reedy, 2017). Such predictions are characteristic of the past decade of AI hype.

A significant number of recent studies have countered this AI hype in fields such as translational medicine (Toh et al., 2019), multidisciplinary medical teams (Di Ieva, 2019), radiology (Rockall, 2020), COVID-19 (Abdulkareem and Petersen, 2021), machine vision (Marquardt, 2020), management (Holmström and Hällgren, 2021), and interaction design (Liikkanen, 2019). The advances of the last decade have indeed been significant, but AI is only capable of performing well on narrow tasks for which they can be trained over an extended period with a significant amount of relevant data (and significant number of humans working on labeling this data). The disconnect between the specialist capabilities of a neural network which has learned a specific task and the general capabilities of AI to perform a range of tasks remains significant.

Maclure (2020) has described the tendency to make unsupported claims about the speed of technological progress as “AI inflationism”. He argues that inflationism focuses our collective ethical energies on the wrong problems. The close attention required to apply a set of abstract ideas to a concrete situation necessarily results in a selective approach. Even an ethics based on the broadest deontological principles becomes selective when those principles must be applied to a particular dilemma: the deontologist answering the trolley problem must necessarily be thinking about the trolley's direction of travel. AI inflationism risks concentrating our ethical energies on issues which are not yet relevant, at the expense of those which are. Inflationist approaches which center ethicists' attention on topics such as the best response to the singularity indirectly prevent us from paying attention to the issues that impinge upon the wellbeing of people who interact with significantly less advanced AI right now.

We draw two lessons from this critique. First, we should avoid expending our finite ethical energy on speculative digressions and ensure that our focus is on applying ethics to the most salient issues. Second we should conduct research into AI on the basis of a fundamental continuity with wider studies of technology in the capitalist workplace. As such, we advocate a deflationary approach which, in line with Maclure (2020), attempts to look past the AI hype to identify the risks and opportunities raised by the current deployment of AI in the workplace.

AI inflationism leads to a perception of technological exceptionalism. Because AI is unlike any previous technology, the thinking goes, all historical ways of thinking about technology are irrelevant. Such exceptionalist narratives can contribute to the degradation of ethical standards in academic AI research. For example, as Metcalf and Crawford (2016) have argued, research that uses large quantities of data in higher education contexts in the US have often lacked the kind of ethical control in place in other disciplines. Despite using datasets generated by human subjects, they are not classified as human subject research—often because the data contained within is publicly available. Whereas, an equivalent study in the social sciences would be required to pass ethical review, no such requirement applies in AI research—partly because of its evolution out of disciplines without such institutions in place (computer science, statistics, etc.). One study claimed to use a neural network to identify gang-related crimes with only four data points (Seo et al., 2018). This neural net was trained on Los Angeles Police Department data, which is heavily influenced by a CalGang database that has since been shown to be both inaccurate and deliberately manipulated by LAPD officers (Davis, 2020). Despite the potential harm caused by a neural network reproducing failures of the database and intensifying the patterns of systematic discrimination present in LAPD policing practices, there was no ethical review of the research. Issues such as bias and racism went completely uncommented upon in the paper itself. As Crawford (2021, p. 116–117) argues, “the responsibility for harm is either not recognized or seen as beyond the scope of the research.” The exceptional framing of AI contributes to the absence of ethical standards.

We have already noted the lack of attention to labor in the literature; AI exceptionalism risks exacerbating this further. Instead, we argue, the long history of thinking about technology in the workplace—from Smith's (1776) *Wealth of Nations* and Ure's (1835) *Philosophy of Manufactures* to Marx [1887] (1976) *Capital* and beyond—has much to tell us about the way in which AI operates in our context today. For example, by emphasizing a continuity-based analysis of technology, Steinberg's (2021) work on the automotive lineage of the platform economy presents an analysis of a supposedly novel technology (labor platforms) that is situated in the historical tendency to outsource labor costs and mine data from labor processes. AI is best understood in context and as a distinct development in a lineage of technology. Rather than being generated *ex nihilo*, AI is a product the same mode of production that gave us the spinning jenny. The social relations that shape AI are familiar to us and existing theories of work technology have much to teach us about AI's development. Analysis must strike a balance between what is old and what is new so that it can accurately represent the degree of continuity and discontinuity in technological change. Ethical approaches which fail to understand this basic point and buy into AI inflationism and exceptionalism tend toward making three kinds of errors: (A) focusing on potential ethical challenges that may arise in the future rather than existing problems of the present, or postponing dealing with the ethical challenges until they become a major problem that cannot be ignored (Ustek-Spilda, 2019); (B) abdicating or deferring responsibility for creating robust ethical standards and regulations due to the perceived speed of AI's development, and the curious assumption of ethics potentially being in conflict with technology development; and (C) failing to see the fundamental continuity of AI with a longer lineage of technological development (Law, 2004) which can help contextualize our current ways of thinking and acting. Hence we argue that a deflationary approach to AI must insist it is *not* exceptional to similar historical waves of technological change, and the continuities between past and present are more important to explore than the unique aspects of AI for developing ethical principles and practices.

### The Problem With Automatic Politics (Ethico-Political Critique)

The drive for accumulation is inherent to capitalism and with it “an autonomous tendency for the productive forces to develop” (Cohen and Kymlicka, 1988, p. 177). How these forces develop in relation to capital's imperative to control them, however, is socially shaped by the regulation of markets, finance, state power, geopolitics and the power of organized labor. As Noble (1984) noted, technologies alone do not determine changes in social relations but rather tend to reflect such changes. There is a dominant view among AI positivists that technological innovation always constitutes social progress. Yet this view ignores the politics of design and production. Sabel and Zeitlin (1985) argue that politics determines technology design and implementation at work, rather than an inherent capitalist drive toward efficiency. At the same time, technology design also tends to require or strongly encourage particular forms of social organization (for a discussion of the machine-determined “intelligence” in AI see Moore, 2020). This tension forms a dialectic that shapes the boundaries of control. The accountability mechanisms (or lack thereof) that stem from a particular politics—whether at the level of the state or the workplace—will ultimately determine the impact of technology design on workers.

This dialectic is observable in the recent emergence of information and communications technologies (ICT). In her example of the introduction of mobile phones for managers, Orlikowski (2007) takes up a soft version of Winner's (1980) thesis that artifacts have politics. Mobile phones did not simply make communication more convenient—they changed that nature of communication itself. Similarly, cloud technologies have not simply enabled greater connectivity, they have changed what connectivity means. Recent legislation such as the “right to disconnect” was introduced in France to limit the political impact of mobile and cloud connectivity on workers, and the push toward always being available for work, blurring the distinction between work and home, private and public sphere and online and offline hours.

The tension between technology development and the politics of the labor process is further illustrated by the “labor extraction problem”, i.e., the broad range of factors employers use to minimize unit labor costs (Edwards and Ramirez, 2016). There is always a trade-off between positive rewards for performance and negative punishments for failing to meet standards—all of which depends on work culture, supervision costs, social protections for workers, and the power of organized labor. Technology doesn't sit independently of these factors; it is always already socially embedded. The ways technologies like AI are developed is inextricably bound with the ways in which companies' direct innovation, diffusion and application to the tasks most attuned to the profitable extraction of surplus-labor. The degree of labor effort is integral to this extraction and requires the development of different organizational strategies. From tightly controlled and fragmented tasks performed on a continuous, mechanized production line to complex, team-driven and capital-intensive production systems—extraction requires different levels of discipline (Edwards and Ramirez, 2016). In complex production systems, the absence of just one worker could disrupt the entire network of labor, thus increasing workers' bargaining power vis-à-vis capital. However, this degree of power may induce management to reduce the complexity of tasks and substitute machinery for labor, depending on the costs and benefits of control. Another factor concerns the costs of work performance monitoring. If it is expensive due to the need for human supervisors and workers are in a strong bargaining position due to labor protection, unions and/or high technical knowledge; employers will tend to use positive incentives to elicit greater labor effort. If, on the contrary, worker performance monitoring is cheap and workers can be disciplined, dismissed and replaced easily (though using self-employment or temporary contracts, for example), then negative incentives will tend to be used (Edwards and Ramirez, 2016).

Digital labor platforms (a prevalent use-case of AI) are illustrative of how this labor extraction takes place. As Stanford (2017) points out, technological changes that do not require large amounts of direct capital investment (such as cloud-based AI-powered platforms), enable the decentralization of production through mobile tracking, surveillance and algorithmic management without necessarily sacrificing the element of direct employer control. For example, platforms such as Uber use facial-recognition AI to verify user identity and rely on customer ratings and real-time movement tracking in their app to manage a global workforce of drivers. Ratings and automated tracking essentially outsource performance monitoring and keep management costs low—yet this has real costs for workers (Moore, 2018). For example, racial bias in facial-recognition AI has led to the deactivation of many non-white Uber drivers, because the technology would not recognize their face (Kersley, 2021). This caused considerable disruption to workers' livelihoods. Similarly, unfair or inaccurate customer reviews can reduce drivers' earning capacity, and in the worst cases lead to deactivation with no opportunity for formal mediation by a trade union (Temperton, 2018). If it is legal to terminate workers' contracts based on an algorithmic decision without any transparency or formal process of contestation, managers can simply defer to the “black box” of the AI system rather than being held to account for the design of such systems themselves.

As noted above, whilst both employers and trade unions might agree on the need for fairness in applications of workplace AI, the question of what each party considers “fair” is likely to differ significantly. Rather than building agreement, such statements of principle simply identify the values over which conflict will take place between actors with opposing interests. One potential solution to this conflict would be for individual stakeholders to produce documents determining the meaning of ethical practice in isolation. IBM's *Everyday Ethics for Artificial Intelligence* (2019), for example, achieves a higher level of concrete detail than we might otherwise expect by using a hypothetical example of a hotel implementing an AI virtual assistant service into its rooms to demonstrate how five particular areas of ethical focus (accountability, value alignment, explainability, fairness and user data rights) might be applied in practice. That said, when it comes to defining what it means by “fairness”, the document only identifies the need to guard against algorithmic bias, ignoring other potential negative impacts such as undermining workers' decision-making capacity, deskilling, or even jobs destruction. Furthermore, broader issues in the sector such as low wages, insecure employment and lack of collective bargaining are not considered, implying that such concerns somehow lie outside the realm of technology ethics.

In this context, we might detour Crawford (2021) notion that “AI is politics by other means” and posit that AI is politics by automatic means. Multi-stakeholder agreements involving high-level principles can hide profound differences in political assumptions and the divergent interests of labor and capital. Without independent accountability mechanisms aimed at more equitable social outcomes, AI will simply deepen existing inequities.

### Big Tech Ethics Is Unilateral

One of the principal issues with AI ethics frameworks is that the development of self-assessment and voluntary guidelines involves a conflict of interest. As Bietti (2019) notes, tech companies tend to deploy ethics frameworks to avoid statutory regulation and serve as a defense mechanism for criticism from wider society. Lack of disclosure, regulation and protection increases the autonomy of capital and increases a range of public threats from automated hacking (Veiga, 2018) to political disinformation and deep fakes (Westerlund, 2019). In this context, self-regulation is a direct attempt to avoid any real accountability to the public and inevitably serves the interests and objectives of capital and companies themselves. External mechanisms are the only way that the public can exercise power over AI companies and hold them to account.

The case of Google is instructive on how ethics unilateralism fails. Since 2017 Google has attempted to implement an AI ethics strategy through top-down internal policies, in response to a backlash of criticism by both Google employees and the public. The backlash was first provoked by the revelation that Google had partnered with the US Department of Defense who were using their TensorFlow AI system in military drone programs known as Project Maven. Numerous other internal strategies were pursued by Google, such as setting up an Advanced Technology External Advisory Council (ATEAC), whose mission was to consider the “most complex changes that arise under (Google's) AI principles” (Google, 2019). It was quickly disbanded a week later as members resigned over the failure of the company to live up to its political principles (Phan et al., 2021). Google persisted in other attempts to facilitate more ethical AI by consulting with academics and community-based, non-profit leaders, and recruiting ethicists as part of the Google Research Ethical AI team (Google, 2020). Yet regardless of how refined and well-considered any resulting principles might be, there is virtually no enforcement, and no consequences for breaching them by any statutory body. Google employees continue to be fired for speaking out against the company (Ghaffary, 2021).

Other voluntarist initiatives aimed at Fairness, Accountability, and Transparency (FAccT) in AI and ML such as AI Fairness 360 by IBM, Google Inclusive ML, and Microsoft FairLearn^3^ have been developed in collaboration with universities (Phan et al., 2021). The development of these products allows firms to claim they have solved the problem of bias and revised their customer-facing brand identity along ethical lines. Yet the development of ethics frameworks through tech-company funded University research projects largely serves the interests of the private sector, and therefore capital. The individualized, privatized, and voluntarist nature of these initiatives also poses a fundamental limit to the scale and scope of enforcement.

Indeed, efforts to debias AI never seem to consider the bias of capital, i.e., the interests of shareholders over workers, accumulation over distribution, and private exchange-value over social use-value. The prevailing restriction of the AI ethics discussion to the classical liberal principles of property and privacy also takes effect in discussions of bias. The issue has primarily been framed as one of poorly trained algorithms acting in a way which illegitimately penalizes individuals or groups. But bias in the form of specific inaccuracies is less concerning than the broader reproduction of existing patterns of social inequality *via* AI (Eidelson, 2021). The majority of AI developed in the private sector has, for instance, systematically biased the interests of shareholders and managers over the interests of workers, and placed the private accumulation of capital over the public accumulation of social goods (Crawford, 2021). Such considerations are (unsurprisingly) not within the remit of Microsoft, IBM, or Google's FAccT programs. This glaring omission highlights how inadequate the self-regulation of such biases will likely prove in the long term. In proposing technological solutions to social problems, these initiatives mask the wider social and economic context in which they are operating. Unilateral ethical commitments tend to avoid the difficult areas where the interests of the party writing that commitment contrast with ethical practice—and therefore fail to address the areas of greatest risk.

The obvious solution is to develop and apply ethical principles through collaborative multilateral processes which involve a variety of stakeholders. Many sets of ethical principles have embedded a commitment to social dialogue, but often this commitment remains largely non-binding and non-specific, and it rarely goes beyond the immediate discursive bubbles of those setting up the dialogue. What is needed, if a set of principles are to actively foster the kind of multistakeholder engagement that can turn ideas in to practice, is a concrete set of accountability and enforcement mechanisms that can allow for negotiated agreement over areas of conflicting interest.

## Ethics vs. Accountability

### Statutory vs. Non-statutory Accountability Mechanisms

It is to this question of accountability mechanisms which we will now turn. Hagendorff (2020) has demonstrated that most of the 100+ ethical AI statements of principle generated in the last decade have had minimal practical impact. Stakeholders who want to support the development of ethical AI therefore face an uphill battle. Our argument is that if the jump from ethical theory to practice is to be successfully made, then the field of AI ethics must progressively replace the dominant pattern of seeking consensus through increased abstraction with negotiating multistakeholder agreements through progressively greater levels of detail.

Regulators and the public are entitled to clear explanations of the rules and choice criteria of AI technologies, despite their proprietary nature, and voluntarist ethical guidelines will be useless if the algorithm remains a black box (Karen and Lodge, 2019). Some claim that the complexity of the technology presents serious barriers to explaining how a particular function was carried out and why a specific result was achieved (Holm, 2019). However, there already exists a mechanism enshrined in workers' statutory rights through which *accountability* (if not explainability *per se*) can be carried out through multi-stakeholder negotiation—collective bargaining. At both the sector and enterprise level, collective bargaining offers stakeholders a way to agree the concrete details of ethical AI implementation in the workplace, with the introduction of new AI ideally being negotiated beforehand, not retrospectively, if optimal translation of principles to practices is to be achieved (De Stefano and Taes, 2021).

Early case studies of how collective bargaining operates to produce ethical outcomes are beginning to emerge. Workers represented by the German union ver.di expressed concerns over the use of RFID technology and algorithmic management by multinational retail corporation H&M. The risk of negative impacts such as deskilling, work intensification, unwarranted increases in managerial control, workforce segmentation, and increases in precarity were significant for them. Using their works council, retail workers were able to delay the introduction of the new technology pending further negotiations over risk mitigation measures (López et al., 2021). Here, the unilateral implementation of new forms of AI in the face of ethical concerns was avoided because collective worker power was exercised to assert co-determination rights.

It is indicative of the managerial bias of the AI ethics literature so far that collective bargaining has rarely been mentioned as an essential part of the translation between theory and practice. But it is by no means inevitable that the representatives of capital should rigidly oppose collective bargaining. Indeed, robust collective bargaining has historically facilitated forms of partnership between labor and capital in Northern European economies. At the level of the firm, it has tended to reduce industrial conflict and employee turnover and increase trust and cooperation. On the national level, it has frequently been one factor in reducing strike rates, increasing productivity, and controlling the pace of wage growth (Doellgast and Benassi, 2014). The desirability of these outcomes for workers themselves is debatable, yet opposition to collective bargaining is by no means a necessary position for the representatives of capital. Any employer seriously interested in the ethical application of AI in the workplace should proactively respect workers' rights to organize and ensure workers' perspectives are represented as far as possible pre-union.

Statutory regulations around the use of technology, including AI, in the labor process have been developed, introduced and enforced in many countries, and this process will gradually see broad theoretical principles about AI ethics translated into legislation. This is to be welcomed, but the ability to introduce and shape legislation tends to be restricted to a small range of actors, locking out many interested parties from direct mechanisms through which they can support that translation process. As a result, there remains a significant need for forms of non-statutory regulation which can be designed and implemented by civil society actors acting outside of (and often in opposition to) governments. For example, the Living Wage Foundation's non-statutory identity was used by the UK Government to market their statutory changes to the minimum wage.

Positive examples of non-statutory regulation are already abundant in the world of work. As shown by the Fairwork project,^4^ objective monitoring of labor standards in the platform economy by researchers can contribute to raising standards across 27 different countries. For example, following low scores for fairness in Ecuador and Ghana, food delivery platform Glovo consulted Fairwork on the creation of a “Courier Pledge” that aimed to introduce a set of basic standards.^5^ Not all of Fairwork's suggestions were implemented, but Glovo *did* introduce a living wage guarantee for all the hours couriers were logged into the app; the provision of health and safety equipment for couriers; the creation of a formalized appeal process for disciplinary action with access to a human representative and a mediator system; a commitment to introduce channels of the improvement of collective workers' voice; and the institutionalization of anti-discrimination policies.

This crisis of ethical impact that Hagendorff (2020) identified is not an inherent feature of AI as a technology. While statutory solutions offer the best accountability mechanisms, there remains a place for non-statutory mechanisms. With the right models for translating principles into practices, there are ways for non-statutory regulation based on statements of ethical principle to shape the way in which AI is implemented in the workplace. In line with our critique above, however, this approach to AI ethics should not just look like a repetition of what has come before. As well as changes to the content of principles, AI ethics should be open to new modes of translation. The example of Fairwork demonstrates that non-statutory regulation will have to be both willing to take a potentially adversarial stance toward AI developers and employers who use their products, while also be willing to prioritize collective worker voice and participation if it is to start forcing profit-motivated private companies to act more in the interests of society at large.

### Proposed AI for Fairwork Standards

We identified the important gap of omitting workplace, employment and labor concerns from AI ethics. We also noted that in order for ethical principles to be implemented into practices, we need the organizations to be not merely committing to them voluntarily, but actually be held accountable to them. Working in partnership with the Global Partnership on AI (GPAI), the authors are involved in an ongoing “AI for Fair Work” project to create a set of principles and an associated non-statutory implementation scheme which can deliver on this goal. The 10 initial principles developed in the project which aim to address the gaps identified in the earlier sections of this paper are summarized in Table 2.

**Table 2:** Nine draft principles for the GPAI's “Fair Work for AI” project.

1	Guarantee decent work	The right to decent work has been extensively established. The introduction of AI to a labor process is no excuse for undermining basic labor standards. We also cannot assume that decent work conditions are going to be provided *de facto* in new working arrangements and can be taken for granted. Regardless of changes in workplace technology, this right must be upheld
2	Build fair supply chains	AI development is not conducted in isolation. The requirement to pursue fair conditions must apply across the supply chain, and organizations have a responsibility to use their procurement power toward that end and should be held accountable of the practices of the parties they subcontract parts of their work
3	Promote explainability	Workers have a right to understand how the use of AI impacts their work. Organizations must respect this right and provide detailed, understandable resources to allow workers to exercise it
4	Strive for equity	The way AI is produced means that it is never purely objective. So, the values used to design AI need to be open for discussion and evaluation with the goal of minimizing both algorithmic bias and patterned inequality
5	Make fair decisions	The automation of decision making can lead to a loss of accountability, but mere human oversight over decision making doesn't guarantee fair decisions either. By combining a strong right of appeal with a process to implement lessons learned, organizations can create a robust system which harnesses the power of AI whilst delivering fairer decisions that take into account limitations to resources and socio-economic opportunities, but aims to reduce injustices in their allocation as much as possible
6	Use data fairly	The concentration of data can create risk both for individual persons and groups, so limits must be put on collection (i.e., data minimization) and processes created for subjects to access their personal data in a comprehensive and explainable format. There should be opportunities for individuals to learn and increase their understanding about potential data risks, so that they are able to question and when necessary, reject, decisions made about them
7	Enhance safety	The right to healthy, safe working environments must be protected. Advances in algorithmic management have increased the risks of work intensification and surveillance. Organizations should seek to actively improve health and safety through their technology
8	Create future-proof jobs	The introduction of workplace AI can cause specific risks such as job destruction and deskilling. These risks can be avoided by treating the introduction of AI as an opportunity to engage in a participatory and evolutionary redesign of work. This approach should mitigate the risks above and look to use the advantages conferred by the use of AI to increase job quality
9	Advance collective worker voice	By facilitating collective bargaining, stakeholders can create the conditions for productive negotiation to determine how to turn ethical principles into ethical practice. This also guarantees the principles to be embraced by a larger group of the society, and the developers and users of AI to be held accountable

The full detail of these principles and their associated measurable benchmarks will be available in a report in 2023, following the conclusion of the consultation process. However, we believe it will be of value to discuss how our critique of the existing AI ethics literature has informed the drafting of these principles, even in advance of the full results of the project being available.

These principles refuse the narrow liberalism inherent to much of current AI ethics debate, which tends to remain in the classical frame of property and privacy. Instead, this project accepts the need to deal directly with the often-suppressed issues of power and control in the workplace. The values encoded in the social relations of production are not an epiphenomenon of ethical discussion that is more properly conducted in the purely conceptual terrain: instead, these values are often determined by the balance of forces between groups of agents and their ability to advance their respective interests. Where the interests of labor and capital do come into conflict, two choices are available: either a retreat toward unilateral principle statements made by individual stakeholders in isolation, or a mechanism to negotiate that conflict in order to achieve improvements in ethical practice.

Collective bargaining is a crucial a mechanism to negotiate the conflict between capital and labor, though it varies hugely across different global contexts. Not only does the absolute number of workers covered by an agreement differ from country to country, so too does the dominant kind of agreement: whilst some cover entire sectors, some are only relevant for specific employers or sites of employment. This diversity necessitates a certain degree of adaptability in how the principles can be applied. As a result, the principles also contain a draft provision for an anonymous consultation process which can be applied in workplaces where there is no trade union presence—whilst emphasizing the need for organizations to respect the right to organize of all workers and not in any way seeking to circumvent union organization. Taken together, these principles attempt to avoid the pitfalls identified in the discussion above and identify a route through which stakeholders can work toward the implementation of fairer workplace AI that mitigates the risks and maximizes the opportunities associated with this ongoing process of technological development.

## Conclusion

To paraphrase James Ferguson in his critique of “development”, what do existing ethical AI principles do besides fail to make AI ethical? It's not just that they are ineffective, it's that they can provide a screen to all manner of unethical behavior and practice. We have argued in this paper that ethics must be focused on the concrete to make them useful. The principles we have presented hone in on the immediate challenge presented by AI in the workplace. In part, the draft of the principles has drawn on pre-existing standards and understandings of rights in the workplace, but it also goes beyond them. The work-centered critique of existing principles and the proposed new standards set outs a research agenda and is the primary contribution of this article to the burgeoning literature on AI and work.

Worker voice has been significantly neglected in debates around AI, and so we have paid particular attention to those critiques leveled from the perspective of workers on hegemonic ethical values as they apply to the workplace. As part of adopting a deflationist attitude to AI, this has often meant looking back at historic theories of technological change. For example, Braverman's analysis of the deskilling tendencies of Taylorism is the major theoretical background to principle nine: increase job quality. This historical perspective also emphasizes the need for stakeholders to begun to formulate rules that govern the operation of technologies before path dependencies can block off potentially emancipatory or liberatory routes for development.

As a result, the principles emphasize the need for external normative values to be imposed on field of possibilities created by tech. This inevitably means that we don't just need workers as stakeholders—we also need governments. The regulatory turn is now well underway with respect to AI, and the end goal of any discussion of normative values must be to feed into that process of development. By involving representatives from global governments in the consultations conduct over the principles, we aim to link these discussions into concrete programs of action at the legislative level.

## Data Availability Statement

The original contributions presented in the study are included in the article/Supplementary Material, further inquiries can be directed to the corresponding author.

## Author Contributions

MC lead the research and writing of the paper and organized the argument, contributed the most time in research and writing, particularly around the literature review, the second two critiques, the abstract and framing, and provided detailed edits and feedback. CC contributed the second most time researching and writing the paper, is the lead in GPAI, and contributed to two seconds as well as writing up the principles which all four authors contributed to developing. FU contributed primarily around ethics questions. MG had the idea to develop the paper and provided editorial and conceptual work. All authors contributed to the article and approved the submitted version.

## Funding

This paper was made possible by funding from the Global Partnership on Artificial Intelligence.

## Conflict of Interest

The authors declare that the research was conducted in the absence of any commercial or financial relationships that could be construed as a potential conflict of interest.

## Publisher's Note

All claims expressed in this article are solely those of the authors and do not necessarily represent those of their affiliated organizations, or those of the publisher, the editors and the reviewers. Any product that may be evaluated in this article, or claim that may be made by its manufacturer, is not guaranteed or endorsed by the publisher.

## Footnotes

^1^https://oecd.ai/en/dashboards.

^2^See https://www.moralmachine.net/.

^3^See IBM https://aif360.mybluemix.net/, Google https://cloud.google.com/inclusive-ml and Microsoft https://www.microsoft.com/en-us/research/uploads/prod/2020/05/Fairlearn_whitepaper.pdf respectively.

^4^https://fair.work.

^5^Conflict of interest statement: None of the researchers have any connection with any of the platforms, the work undertaken received no funding or support in kind from any platform or any other company, and we declare that there is no conflict of interest.
