# Supplementary material for: Politics by Automatic Means? A Critique of Artificial Intelligence Ethics at Work
Source: Front Artif Intell. 2022 Jul 15;5:869114. doi: 10.3389/frai.2022.869114 (PMC9334705; doi:10.3389/frai.2022.869114)

## Appendix

SCOPUS Search

"AI ETHICS" AND work* AND ( LIMIT-TO ( DOCTYPE , "ar" ) OR LIMIT-TO ( DOCTYPE , "ch" ) OR LIMIT-TO ( DOCTYPE , "bk" ) ) AND ( LIMIT-TO ( LANGUAGE , "English" ) ) AND ( LIMIT-TO ( PUBSTAGE , "final" ) )

[Date 18 Nov 2021]


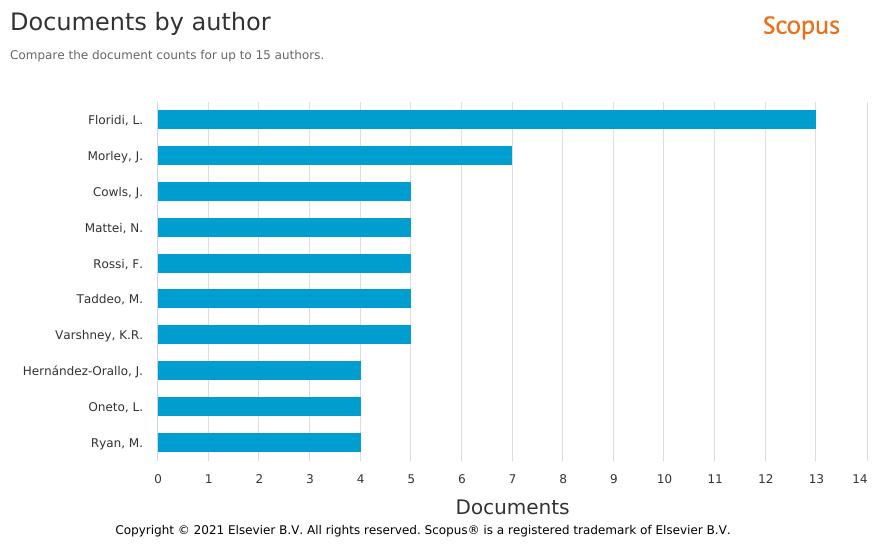


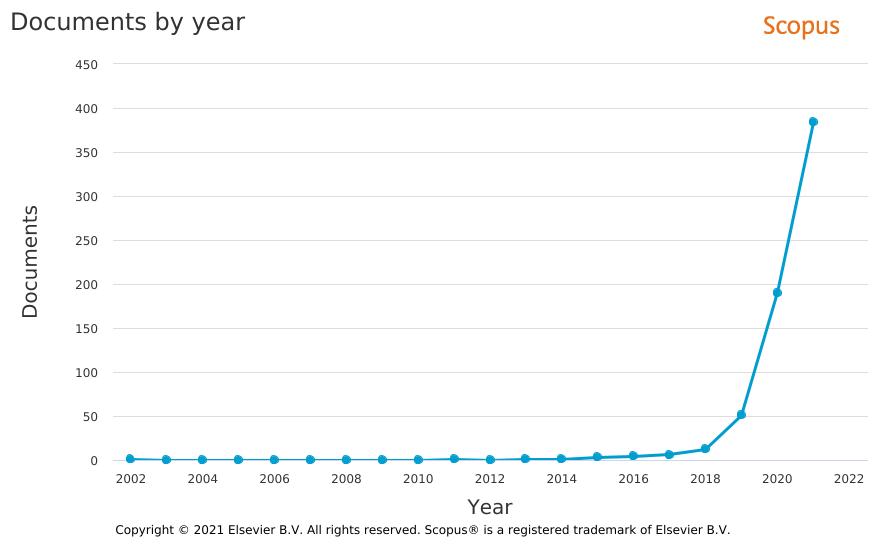


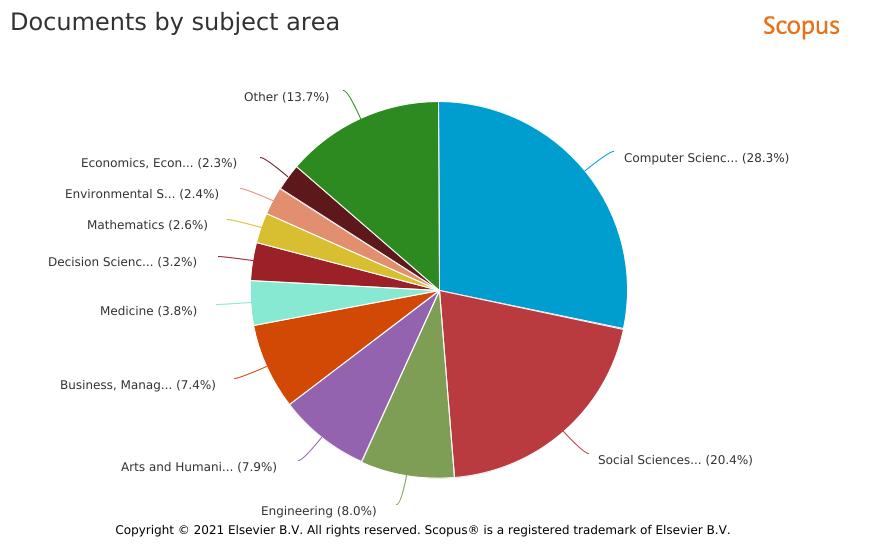

Supplement: Supplementary file 1 [file Data_Sheet_1.docx]
